# Carbon metabolic versatility underpins *Bathyarchaeia* ecological significance across the global deep subsurface

**DOI:** 10.1093/ismejo/wraf259

**Published:** 2025-11-21

**Authors:** Jialin Hou, Chen Yang, Fengping Wang

**Affiliations:** State Key Laboratory of Submarine Geoscience; Key Laboratory of Polar Ecosystem and Climate Change, Ministry of Education; Shanghai Key Laboratory of Polar Life and Environment Sciences; and School of Oceanography, Shanghai Jiao Tong University, 1954 Huashan Road, Shanghai 200030, China; State key Laboratory of Microbial Metabolism and School of Life Science and Technology, Shanghai Jiao Tong University, 800 Dongchuan Road, Shanghai 200240, China; State Key Laboratory of Submarine Geoscience; Key Laboratory of Polar Ecosystem and Climate Change, Ministry of Education; Shanghai Key Laboratory of Polar Life and Environment Sciences; and School of Oceanography, Shanghai Jiao Tong University, 1954 Huashan Road, Shanghai 200030, China

**Keywords:** archaeal *bathyarchaeia*, carbon metabolism, lignin degradation, deep biosphere, life-Earth co-evolution

## Abstract

*Bathyarchaeia*, among the most ancient and abundant microbial lineages on Earth, dominate diverse anoxic subsurface ecosystems and play a pivotal role in global carbon cycling. This review synthesizes current understanding of their physiological, metabolic, and evolutionary foundations underlying their ecological significance and environmental effects over geological timescales. Despite their global distribution in the deep biosphere, the phylogenetic diversity and total cellular abundance of *Bathyarchaeia* remain substantially underestimated. As uncultivated metabolic generalists, *Bathyarchaeia* exhibit remarkable metabolic versatility, including anaerobic organic degradation, dark carbon fixation, and putative methane and alkane metabolism. Specifically, genus *Baizosediminiarchaeum* has been demonstrated to adopt organomixotrophy by coupling anaerobic lignin degradation with inorganic carbon assimilation. These metabolic strategies likely enable them to thrive in energy-limited subsurface environments with dynamic geochemical fluctuations. The early evolutionary history of *Bathyarchaeia* appears closely linked to major geological events, including tectonic activity and plant evolution, whereas more recent lineage expansions reflect physiological adaptations to host-associated and anthropogenically influenced environments, highlighting their ongoing co-evolution with Earth’s modern environments. Overall, we propose carbon metabolic innovation as the central driver behind the ecological and evolutionary significance of *Bathyarchaeia*, putatively linking microbial ecological functions to planetary biogeochemical processes. Future efforts in isolation and cultivation remain essential for elucidating their unknown physiological and metabolic mechanisms. In parallel, advances in ecological modeling and the development of lineage-specific lipid biomarkers hold great promise for quantifying their contributions to global carbon budgets and reconstructing paleoenvironmental and paleoclimate conditions.

## Graphical Abstract

**Graphical Abstract:**
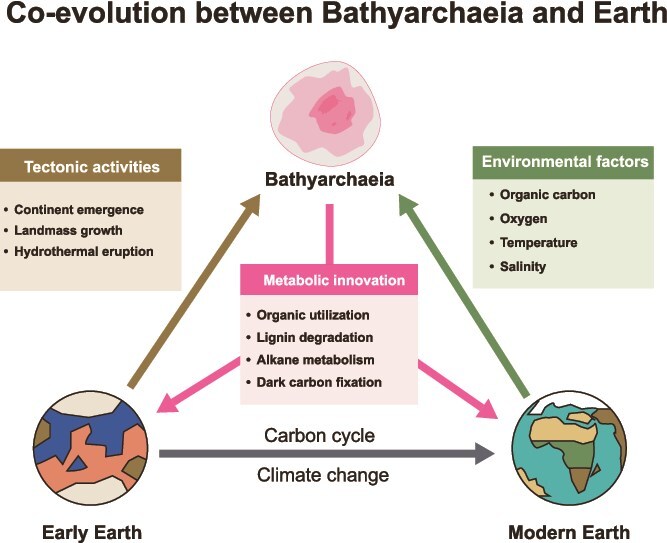

## Introduction

*Bathyarchaeia*, previously known as Miscellaneous Crenarchaeotic Group (MCG) [1] or *Bathyarchaeota* [2], represent one of the most widespread and abundant microbial lineages on Earth [3]. Its members are widely present in diverse anoxic subsurface habitats including marine and freshwater sediments [4], soils [5], hot springs [6], hydrothermal vents [7] and cold-seeps [8], frequently dominating local microbial communities [9]. Despite its ecological prevalence, no pure culture or isolate of *Bathyarchaeia* has been obtained to date. Current understanding thus relies largely on culture-independent techniques, which have revealed their remarkable metabolic flexibility, including anaerobic organic degradation [10, 11] (e.g. lignin [12] and its derivatives [13]), CO_2_ fixation [14] and putative methane/alkane oxidation [15]. These metabolic traits underpin the importance of *Bathyarchaeia* in global carbon cycling. As one of the oldest archaeal lineages, *Bathyarchaeia* is estimated to have originated ~3.3 billion years ago [16], possibly in conjunction with the emergence of the first continental crust [17]. Its evolutionary history may thus reflect and have helped shape early Earth’s environments [17]. Over the past decade, progress in sampling, enrichment strategies, genomic sequencing, and bioinformatic analyses has greatly advanced our understanding of this archaeal lineage. In this review, we synthesize current knowledge on the taxonomy, distribution, metabolism, and evolution of *Bathyarchaeia*, and examine the potential mechanisms behind its long-term ecological significance in the deep subsurface.

## From MCG to *Bathyarchaeia*: a shifting taxonomic landscape

**Figure 1:**
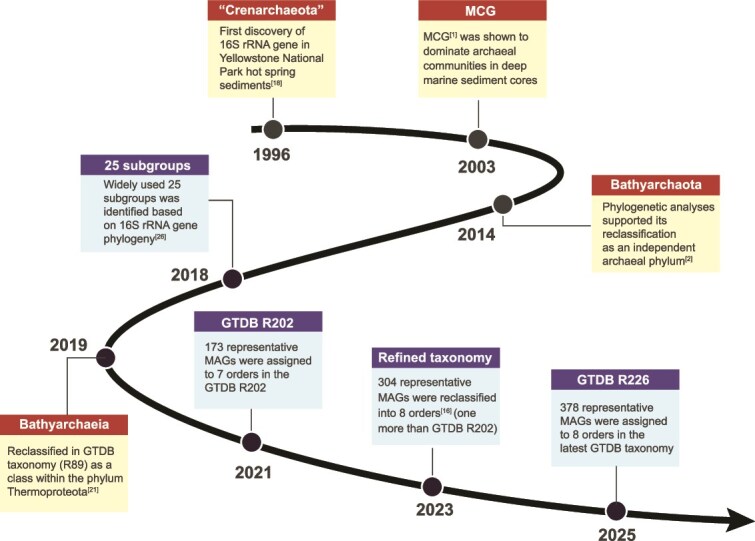
Timeline of developments in nomenclature and taxonomy of *Bathyarchaeia*. Red boxes indicate seminal publications [1, 2, 18, 21] that proposed or revised the nomenclature and taxonomic rank of *Bathyarchaeia*; blue boxes represent selected studies (events) that refined their taxonomic diversity [16, 26]. The GTDB taxonomy (https://gtdb.ecogenomic.org/) has been updated to R226 in April 2025. MCG, Miscellaneous Crenarchaeotic Group; GTDB, Genome Taxonomy Database.

The discovery and taxonomic development history of *Bathyarchaeia* in the past three decades of scientific study is illustrated in Fig. 1. This archaeal lineage was first identified in the early 1990s through 16S rRNA gene sequences recovered from Yellowstone National Park hot spring sediments and initially classified within the phylum *Crenarchaeota* [18]. Subsequent investigations under the International Ocean Drilling Program (IODP) revealed their high prevalence and frequent dominance in deep marine sediments [1], extending to depths of ~86 meters below the seafloor [19]. Additional detections in deep gold mines [20] further underscored their ubiquity across diverse subsurface environments, leading to their provisional designation as the MCG [1] (Fig. 1). Later phylogenetic analyses demonstrated that MCG constitutes a deeply branching lineage distinct from other archaeal phyla, prompting its reclassification as an independent phylum, namely *Bathyarchaeota* [2] (Fig. 1). This classification was adopted by the NCBI taxonomy and has since been widely used in microbiology and microbial ecology. More recently, the Genome Taxonomy Database (GTDB), implementing a standardized, genome-based classification system, reassigned *Bathyarchaeota* to the class *Bathyarchaeia* divided into eight orders within the phylum *Thermoproteota* [21] (Fig. 1). This revision does not diminish the taxonomic significance of this lineage; rather, it positions *Bathyarchaeia* within a rigorous phylogenetic framework that highlights its rank equivalence to other major archaeal groups (such as the phyla *Thaumarchaeota* and *Crenarchaeota* in NCBI taxonomy) and reaffirms its status as a high-rank taxon within the Archaea. Ongoing discoveries of new lineages, e.g. the recently proposed order *Mazuousiales* [16], are expected to further expand the taxonomic diversity of *Bathyarchaeia*.

Due to the absence of cultured representatives, the internal classification of *Bathyarchaeia* has historically relied on the 16S rRNA gene phylogeny—a practice originating with its initial designation as MCG. The early study proposed four subgroups (MCG-1 to -4) [22], alongside several unclassified lineages [23]. Later, the first comprehensive phylogenetic analysis using long 16S rRNA gene sequencing further delineated 18 subgroups (MCG-1 to -17, with divergent MCG-5a and -5b) [24]. This framework was subsequently expanded to 21 subgroups with the addition of MCG-18, -19, and -5bb [4, 25]. By integrating these results with the SILVA SSU 128 database, an important study identified four additional subgroup-20 to -23, establishing a widely adopted taxonomic framework of 25 subgroups for *Bathyarchaeota* [26] (Fig. 1). However, “subgroup” is an operational unit rather than a formal taxonomic rank, defined solely by the 16S rRNA gene phylogeny under variable identity thresholds. For example, 173 representative *Bathyarchaeia* metagenome-assembled genomes (MAGs) in the GTDB archaeal taxonomy (R202) are assigned to 56 genera, 17 families and seven orders (Fig. 1), yet the correspondence between these GTDB ranks and traditional subgroup nomenclature remains unclear. To address this, a recent study refined the GTDB taxonomy of class *Bathyarchaeia* by linking MAGs to subgroups via 16S rRNA gene phylogenies [16] (Fig. 1). The results indicate that these Bathy subgroups are not taxonomically equivalent and thus unsuitable for direct ecological comparisons. Advances in sequencing and bioinformatic technologies, particularly for reconstructing complete genomes directly from environmental samples, are expected to further resolve the phylogenetic diversity and refine the taxonomic resolution of this ecologically significant archaeal lineage.

### From prevalence to predominance: one of Earth’s most abundant archaeal lineages

**Figure 2:**
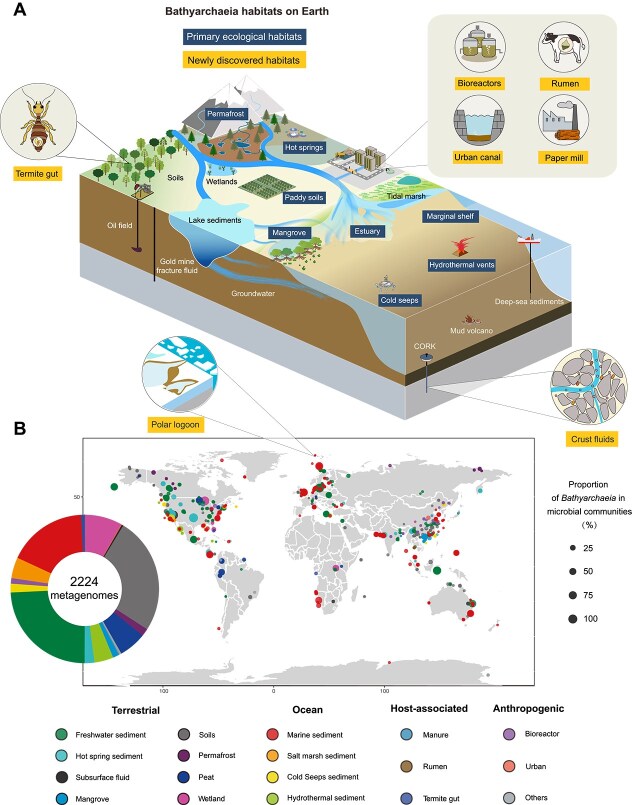
Ecological and geographic distribution of *Bathyarchaeia*. (A) The major habitats of *Bathyarchaeia* on Earth. Dark blue indicates primary habitats where *Bathyarchaeia* frequently dominated; yellow highlights these newly discovered habitats; other habitats are black and white. (B) Geographic distribution and relative abundance of *Bathyarchaeia* across 2244 public metagenomes. Relative abundances of *Bathyarchaeia* were calculated using singleM and Sandpiper [57] (https://sandpiper.qut.edu.au/). All geographic locations and ecological types were manually checked and integrated into major ecological categories; detailed habitat classifications are provided in Supplementary Table S1.

*Bathyarchaeia* are ubiquitous in diverse subseafloor environments, including intertidal zones [12], estuaries [27], salt marshes [28], mangroves [29], and deep sedimentary cores [30] (Fig. 2A). They thrive particularly in organic-rich anoxic marine sediments [25], where *Bathyarchaeia* frequently dominate archaeal communities, constituting ~30–60% of archaeal populations [30] and accounting for up to 90% in some cases [10]. A recent global survey further reinforces their widespread occurrence and quantitative dominance in marine sediments across the globe [9], even in the deepest hadal zone, Mariana Trench [31] (also see Fig. 2B). In this context, continental margin sediments were estimated to harbor ~2–3.9 × 10^28^  *Bathyarchaeia* cells, corresponding to ~7%–13% of the total subseafloor microbial population [3]. This estimation, however, did not account for deep-sea subseafloor microbial hot spots, such as hydrothermal fields [32] and cold seeps [8], where *Bathyarchaeia* are often highly enriched. Furthermore, MAGs recovered from anoxic crustal fluids in boreholes drilled into Juan de Fuca Ridge flank indicate their presence in the deep basaltic environment [33]. Taken together, the total cell number of *Bathyarchaeia* in global marine subsurface environments is likely much higher than previously estimated.

Beyond marine environments, a variety of terrestrial subsurface habitats have emerged as significant reservoirs for *Bathyarchaeia* [16, 34], particularly waterlogged anoxic sedimentary settings, such as paddy soils [35], wetlands [29], deep lacustrine settings [36, 37], lagoons [38], hot springs [6], peatland [39] and permafrost [40] (Fig. 2A). For example, they are widely distributed in global paddy soils, averaging ~32% of archaeal communities and reaching up to 90% in some cases [35] (Fig. 2B). Tropical peatlands also harbor high abundances of *Bathyarchaeia*, up to 80% of the archaeal communities in the deep samples [5]. Furthermore, recent studies have identified termite [41] and certain vertebrate guts [42] as previously overlooked host-associated habitats for these archaea (Fig. 2A). Given that the global continental subsurface harbors ~2–6 × 10^29^ microbial cells [43], the total population of terrestrial *Bathyarchaeia* cells is likely comparable to, or may even exceeds, that in marine sediments. Therefore, the quantitative predominance of *Bathyarchaeia* across both terrestrial and marine deep subsurface environments suggests that their global population size substantially exceeds the earlier estimate of ~2–3.9 × 10^28^ cells [3]. Consequently, *Bathyarchaeia* may represent one of the most abundant microbial lineages on Earth, particularly in anoxic niches, highlighting their successful evolutionary history and significant roles in linking marine and terrestrial biogeochemical cycles.

### From salinity to temperature: key drivers of biogeographic distribution

As one of the most successful microbial generalists on Earth, *Bathyarchaeia* inhabit ecosystems spanning a broad spectrum of physicochemical conditions (e.g. temperature, oxygen level, salinity, and others), from polar lagoons [38] to hot spring sediments [6], and from paddy soils [35] to hypersaline deposits [28] (Fig. 2). Beyond natural environments, several *Bathyarchaeia* lineages have also been detected in host-associated and anthropogenic settings, such as termite guts [41] and urban canals [44]. This broad biogeographic distribution and wide niche breadth reflect not only their evolutionary success but also lineage-specific adaptations to diverse extreme environments, underscoring their value as a model for studying environmental adaptive mechanisms in Archaea. Recently, the identification of butanetriol dialkyl glycerol tetraethers (BDGTs) as a lineage-specific lipid biomarker for genus *Baizosediminiarchaeum* suggests that unique membrane lipid composition could be a key adaptive trait facilitating its prevalence in marine sediments [45].

Environmental parameters play a key role in structuring *Bathyarchaeia* communities, with salinity emerging as a major driving factor of taxonomic diversification [4]. It clearly distinguishes terrestrial from marine communities: the genus *Baizomonas* (comprising former subgroups-6 and -5bb) predominates in global paddy soils and accounts for over 90% of *Bathyarchaeia* in global paddy soils [35], whereas marine and saline sediments host more diverse assemblages, often dominated by the genus *Baizosediminiarchaeum* (previously subgroup-8) [45]. Furthermore, salinity gradients significantly influence *Bathyarchaeia* diversity in estuarine systems, with remarkable community composition shifting between freshwater and marine conditions [27], indicating distinct osmoregulation mechanisms likely enable their persistence across different saline environments.

Oxygen availability also critically shapes the distribution of *Bathyarchaeia*. Although most known lineages are anaerobic and prevail in anoxic habitats [9] (Fig. 2A), accumulating evidence indicates that certain groups can tolerate—or even utilize—oxygen in suboxic conditions [46]. For example, some lineages of the genus *Baizomonas* possess genes for oxygen-dependent metabolism, correlating with their occurrence in suboxic surface sediments [47] and microoxic paddy soils [48]. Similarly, the soil-specific genus *Houtuousia* encodes enzymes for the aerobic glyoxylate cycle and cytochrome c oxidases [16], suggesting metabolic adaptation to oxic conditions and a potentially microaerophilic lifestyle. In addition, similar to the *Chloroflexota* lineages in ferruginous sediments [49], antioxidation mechanisms against reactive oxygen species (ROS) is likely an alternative strategy for Bathyarchaeia to tolerate oxygen fluctuations. A recent large-scale metagenomic analysis of the microbiome in Mariana Trench sediments further revealed that microorganisms have evolved two strategies: streamlined and versatile, to adapt to extreme hadal conditions, with antioxidation mechanism playing a central role in coping with extreme hydrostatic pressure [31]. These findings reveal a previously underappreciated metabolic plasticity and adaptability in *Bathyarchaeia*, which may facilitate their niche expansion into suboxic or even oxic environments. Further in-depth investigations are essential to elucidate their ecological trajectory and adaptive strategies under multiple environmental stresses, and to determine whether common adaptation strategies underlie microbial adaptation across different physiochemical conditions [31, 50].

High organic carbon availability characterizes the most known *Bathyarchaeia* habitats, including recently identified anthropogenic settings, such as urban canals [44] and paper mill digesters [51], as well as animal digestive systems like termite guts [52] and cow manure [53] (Fig. 2A). These niches are typically rich in plant-derived organic matter, particularly lignin and lignocellulose, consistent with genomic and incubation-based evidence for the organotrophic degradation of complex organic compounds by *Bathyarchaeia*. In marine sediments, *Bathyarchaeia* abundance correlates strongly with total organic carbon [54], particularly with humic-like compounds [55], underscoring their key role in recalcitrant organic carbon remineralization in the subseafloor. In contrast, *Bathyarchaeia* are detected in oligotrophic open-ocean sediments with relatively limited distribution and abundance, where low availability and high oxidative state of organic carbon likely restrict their colonization.

*Bathyarchaeia* inhabit environments spanning temperatures from >85°C in hot springs [6] and hydrothermal sediments [7] to <0°C in polar lagoons [38], reflecting their substantial lineage-specific thermal adaptation. For example, orders *Bifangarchaeales* (order B24 in GTDB), *Zhuquarculales* (order EX4488-135), and *Jinwuousiales* (order B25), predominantly found in geothermal systems [16], encode heat shock proteins and enhanced DNA repair mechanisms [56], supporting their putative thermophilic or hyperthermophilic lifestyle. In contrast, *Bathyarchaeia* communities in paddy soils display distinct biogeographic patterns in response to mean annual temperature. The dominant genus *Baizomonas*, for instance, shows higher prevalence in moderate temperatures (10–20°C) than in colder (<10°C) or warmer (>20°C) ranges [35]. This suggests thermal constraints potentially limit its distribution in both cold deep-sea and hot geothermal settings. Collectively, temperature critically shapes the biogeographic distribution, evolutionary diversification, and ecological functioning of *Bathyarchaeia* in subsurface environments. Given the central role of archaeal membrane lipids in thermal adaptation and wide growth temperature range of *Bathyarchaeia* [45], future research elucidating the lipid composition and structural modifications in *Bathyarchaeia* is essential to unravel their survival strategies across extreme thermal gradients.

### From methane to lignin: carbon metabolic versatility and innovation

**Figure 3:**
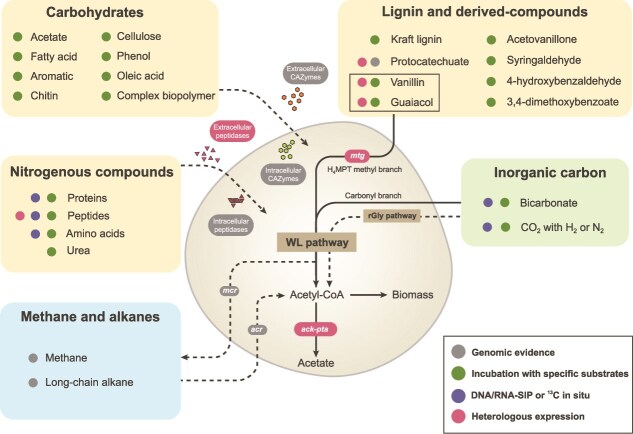
Metabolic capability and potential of class *Bathyarchaeia*. The figure illustrates the metabolic versatility of *Bathyarchaeia* in organotrophic (yellow box), lithotrophic (green box), and methane/alkane metabolism (blue box). Their metabolic substrates are demonstrated by multiple lines of evidence as indicated by coloured dots: green dots, substrates shown to stimulate cell growth [12, 13, 46, 59–61, 67]; blue dots, substrates assimilated in DNA-/RNA-stable isotope probing (SIP) experiments [11, 14, 58, 63]; grey dots, presence of functional genes in genomes/MAGs [2, 3, 15, 16, 69, 72, 78]; red dots, substrates linked to biochemically validated enzymes [2, 3, 10, 66]. Key enzyme abbreviations: mtg, the special methoxyl methyltransferase for *O*-demethylation discovered in *Bathyarchaeia* [66]; ack, acetate kinase; pta, phosphate acetyltransferase; mcr, methyl-coenzyme M reductase; acr, alkyl-coenzyme M reductase; rGly, reductive glycine pathway.

Versatile organotrophy anchored by anaerobic lignin degradation. *Bathyarchaeia* are renowned for their exceptional organotrophic versatility in utilizing a wide range of organic substrates under anoxic conditions (Fig. 3). Early archaeal lipid isotopic tracing suggested that they assimilate organic carbon in deep marine sediments [58]. Subsequent evidence from stable isotope probing (SIP) incubations confirmed that coastal *Bathyarchaeia* could metabolize diverse organic substrates [59, 60] (e.g. acetate and lipids), nitrogenous substrates [10, 28] (e.g. proteins and urea), and complex biopolymers [28, 46, 61, 62] (e.g. oleic acid, phenol, cellulose and particulate algal organic matter). Large-scale MAG analyses further revealed that most *Bathyarchaeia* lineages encode diverse carbohydrate-active enzymes (CAZymes) targeting plant- and biomass-derived polysaccharides, establishing carbohydrate utilization as a core metabolic feature across this class [16]. Specifically, thermophilic order *Bifangarchaeales* from hot spring sediments harbor more CAZyme genes per genome than other orders [16], underscoring their specialized role in carbon cycling of geothermal ecosystems.

Protein degradation represents another widespread organotrophic trait for *Bathyarchaeia*, particularly in the order *Wuzhiqibiales* (previously subgroup-15; order TCS64 in GTDB) [16], which frequently dominate estuaries and marine sediments. Early single-cell genomic and enzymatic evidence first demonstrated that this lineage employs extracellular peptidases to degrade detrital proteins in marine sediments [10], a capability later validated by RNA- and lipid-SIP experiments showing ^13^C-labeled protein incorporation into their biomass [11, 63]. Comparative genomic analysis further revealed that order *Wuzhiqibiales* encodes more peptidase genes per genome than other orders [16], highlighting its specialized niches in protein degradation. As detrital amino acids constitute 67% of chemically characterized sedimentary organic matter [65], protein degradation is a relatively common capability for both bacteria and archaea in marine sediments. However, *Bathyarchaeia* may have specific substrate preference in utilizing proteinaceous compounds, e.g. some lineages were found to actively metabolize D-amino acids in cold seep sediments [64], implying *Bathyarchaeia* play significant roles in protein remineralization and nitrogen cycling in the subseafloor environment.

The utilization of recalcitrant plant-derived polymers, particularly lignin, represents a defining metabolic trait with ecological significance for *Bathyarchaeia* (Fig. 3). Lignin, as Earth’s second most abundant biopolymer, is notoriously resistant to biological deconstruction due to its highly complex, cross-linked methoxylated aromatic architectures [65]. Although lignin depolymerization is traditionally attributed to aerobic fungi and bacteria, a pioneering study demonstrated tenfold growth stimulation of *Bathyarchaeia* (dominated by genus *Baizosediminiarchaeum*) by alkaline lignin under anoxic conditions [12]. This enabled enrichment of Candidatus *Baizosediminiarchaeum ligniniphilus*, the first archaeon utilizing lignin as the primary carbon source [66]. Similarly, another *Baizosediminiarchaeum* strain M17CTs was cultivated on lignin-derived methoxylated aromatics (3,4-dimethoxybenzoate and vanillate) [67], and genus *Baizomonas* was enriched with aromatic aldehydes from lignin (syringaldehyde, 4-hydroxybenzaldehyde, and vanillin) [13]. Both of these genera belong to the order *Baizomonadales* and frequently dominate lignocellulose-enriched sedimentary niches (e.g. estuaries [27] and paddy soils [35]), thereby establishing anaerobic lignin degradation as the core metabolic capability for their ecological success in these environments.

Multi-omics and enzymatic analysis of *Bathyarchaeia* enrichments revealed a unique *O*-demethylation methyltransferase system for anaerobic lignin degradation [66], exclusively conserved in the class *Baizomonadales*. This special system demethylates aromatic methoxy groups and transfers methyl groups to tetrahydromethanopterin (H₄MPT) for acetyl-coenzyme A synthesis. Given that methoxylated aromatics constitute 1.24–24.1% of lignin carbon [65], *Bathyarchaeia* are expected to substantially contribute to lignin mineralization in anoxic marine sediments. Beyond *O*-demethylation, genomic analysis identified key genes for aromatic degradation [68] in *Bathyarchaeia* genomes (e.g. 4-carboxymuconolactone decarboxylase [2] and benzoyl-CoA reductase [69]). However, such genes for canonical aromatic catabolism are absent in the lignin-degrading Ca. *B. ligniniphilus*, implying unknown anaerobic aromatic cleavage mechanisms in this archaeal lineage.

## Overlooked dark carbon fixation in the deep subsurface

Although primarily characterized as heterotrophs, *Bathyarchaeia* also possess the autotrophic capability of assimilating inorganic carbon. Early clues emerged from the detection of ^13^C-depleted BDGTs in estuarine sediments [70], which was recently verified as the lipid biomarker of a dominant *Baizosediminiarchaeum* lineage in global marine sediments [45]. Subsequent lignin-amended SIP enrichment experiments demonstrated the enhanced ^13^C-bicarbonate assimilation into the lipids and DNA of enriched *Baizosediminiarchaeum* [12, 14]. This finding indicates this lineage has a mixotrophic lifestyle, which was later corroborated by another independent DNA-SIP study [63]. Genomic and transcriptomic analyses support a metabolic model in which methyl groups from lignin demethylation and CO₂ are assimilated via the Wood–Ljungdahl pathway (WLP) to synthesize acetyl-CoA, subsequently further directed to biomass synthesis and/or fuel other heterotrophic microbes as acetogens [66]. A further SIP experiment employing gradient ^13^C-bicarbonate labeling demonstrated that *Baizosediminiarchaeum* incorporates carbon into its lipid BDGTs from both inorganic carbon and lignin in an approximate ratio of 2:1 [45]. Collectively, these findings indicate that *Baizosediminiarchaeum*, which predominates in marine sediments, very likely adopts a mixotrophic lifestyle. It underscores the previously overlooked role of *Bathyarchaeia* in the dark carbon fixation and carbon cycling below the seafloor.

Besides the WLP, the reductive glycine (rGly) pathway, recently identified as the seventh carbon fixation pathway in nature [71], has been detected in some *Bathyarchaeia* MAGs with a complete gene repertoire for the serine-bypass variant [16]. This pathway reduces CO_2_ via the methyl branch of WLP and condenses with the second CO_2_ to synthesize glycine via the glycine cleavage system for biosynthesis [71], and thus probably functions as both an auxiliary autotrophic route and redox sink to dissipate excess reducing equivalents [71]. Therefore, the presence of multiple inorganic carbon assimilation strategies in *Bathyarchaeia* underscores their metabolic flexibility and their underestimated role in dark carbon fixation.

## Unresolved roles in methane and alkane metabolism

Over the past decade, one of the most intriguing discoveries regarding *Bathyarchaeia* is their putative methane/multi-carbon alkane metabolism. The initial evidence emerged from two MAGs recovered from the deep coal-bed formation water [72], which encode key genes for the methyl–coenzyme M reductase (MCR) complex (*mcrABG*) and associated methyltransferases. This study hypothesized these enzymes might support their methylotrophic methanogenesis using a variety of methylated compounds [72]. However, subsequent phylogenetic analysis revealed that these *Bathyarchaeia*-type “MCRs” share <60% amino acid similarity with those canonical MCRs involved in methanogenesis or anaerobic oxidation of methane (AOM) [15]. Instead, they are phylogenetically assigned to alkyl-coenzyme M reductases (ACRs) that have recently been found in the long-chain alkane (C ≥ 13)-oxidizing Ca. *Methanoliparum* [73] and hexadecane (C = 16)-oxidizing Ca. *Melinoarchaeum fermentars* [74]. Broader metagenomic survey further indicated that these *Bathyarchaeia*-type ACRs are widespread across deep subsurface ecosystems [75], particularly hot spring sediments, suggesting their potential capability for long-chain alkane oxidation in terrestrial subsurface environments.

More recently, a third, phylogenetically distinct canonical *mcr* gene cluster was identified in a *Bathyarchaeia* MAG [16]. This *mcr* operon phylogenetically clusters with those newly-isolated methyl-reducing methanogens from the phylum *Thermoproteota* [76, 77], suggesting the potential role of *Bathyarchaeia* in methane metabolism, besides alkane metabolism. Although direct experimental validation for these ACR and MCR complexes in *Bathyarchaeia* is lacking, growing evidence from functionally characterized homologues in other archaea strongly suggested that certain *Bathyarchaeia* lineages may mediate anaerobic alkane oxidation or methanogenesis. Given their predominance in anoxic subsurface habitats, *Bathyarchaeia* likely play a previously overlooked role in global hydrocarbon and carbon cycles.

Overall, the metabolic versatility of *Bathyarchaeia* is fundamental for their widespread distribution in global subsurface environments, where effective metabolic innovation, especially in carbon source utilization, might be the driving force of evolution for Bathyarchaeia, facilitating their ecological significance and environmental effect on the Earth system.

### From hot origin to co-evolution: an evolutionary trajectory of life-Earth interactions

**Figure 4:**
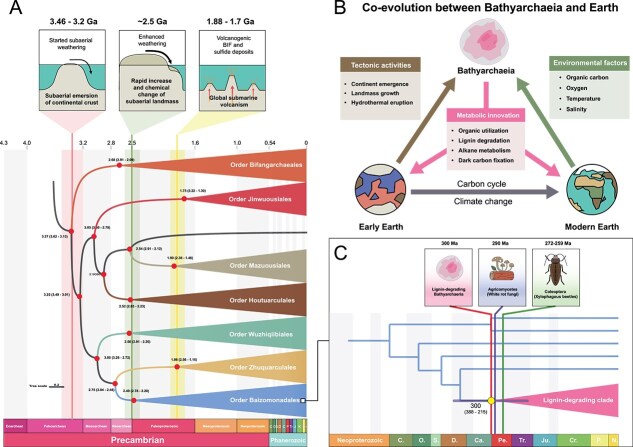
A proposed model for *Bathyarchaeia*-Earth system co-evolution. (A) Evolutionary timeline of *Bathyarchaeia* cladogenesis correlated with planetary-scale geological events: pink column, emergence of the first subaerial continents; green, rapid landmass expansion; yellow, global submarine volcanism. The lower-right panel (C) shows the proposed evolutionary timeline of the lignin-degrading *Bathyarchaeia* lineage (order *Baizomonadales*), alongside the origin of white-rot fungi and xylophagous beetles (Adapted from [16]). (B) Synthetic model illustrates the interactions among *Bathyarchaeia*, early Earth tectonics and modern environments, highlighting the central role of carbon metabolic innovation in driving the life-Earth co-evolution.

As one of the most ancient archaeal lineages, the latest common ancestor of *Bathyarchaeia* is estimated to have arisen ~3.3 billion years ago (late Paleoarchean) [16] (Fig. 4A). Multiple independent phylogenomic and phylogenetic analyses consistently place these lineages from hydrothermal and hot spring sediments, including orders *Bifangarchaeales* (formerly subgroup-21) and *Jinwuousiales* (formerly subgroup-20 and -22), at the base of the *Bathyarchaeia* phylogeny [6, 16, 56]. These findings support the “hot origin” of *Bathyarchaeia* on the early Earth. However, whether this origin occurred in ancient marine hydrothermal vents or terrestrial hot springs remains unresolved. Although 16S rRNA phylogenies suggest a saline, marine volcanic sediment origin [4], the earliest diverging groups (e.g. *Bifangarchaeales* and subgroup-23) are predominantly recovered from terrestrial hot springs [6, 16], implying a possible nonmarine geothermal genesis. A plausible scenario is that *Bathyarchaeia* originated in either shallow submarine volcanic sediments or terrestrial hot springs during the Archean, both environments are known to host Earth’s earliest microfossils [79]. Expanding sampling across geothermal systems and advances in phylogenomic methods will be essential to resolve this early evolutionary history of *Bathyarchaeia*, as well as to elucidate the emergence of life on the Archean Earth.

Over geological timescales, the diversification of *Bathyarchaeia* appears closely linked to major tectonic and environmental transitions (Fig. 4A). First, the divergence of their last common ancestor around 3.3 Ga coincides with the subaerial emergence of Earth’s first continent (~3.46–3.2 Ga) [16, 17]. These nascent landmasses—devoid of plant roots and subject to high tidal fluxes—were colonized by ancient phototrophic microbial mats [80]. These processes enhanced crust weathering and fluvial transport, thus increasing delivery of organic matter and nutrients to coastal zones, forming organic-rich anoxic sediments in early shelf and estuarine settings [81]. Within these habitats, ancestral Bathyarchaeia likely evolved the innovative heterotrophic capacity to utilize microbial necromass (e.g. proteins and amino acids)—a central metabolic trait retained by their modern lineages. The rise of anaerobic heterotrophic *Bathyarchaeia* may have marked the formation and expansion of the anoxic deep biosphere during the Archean, analogous to contemporary subsurface sedimentary environments. Subsequently, several major diversifications of *Bathyarchaeia* were likely influenced by contemporaneous continental growth [82], subaerial exposure [83], and submarine volcanism [84] (Fig. 4A), which collectively reshaped Earth’s subsurface redox state and organic carbon burial patterns, thus creating new ecological opportunities for their metabolic innovation and niche specialization.

The coincident emergence of white-rot fungi [85] and the lignin-degrading *Bathyarchaeia* lineage [12, 66] at ~290–300 Ma [16] implies that their capabilities to decompose this recalcitrant polymer likely evolved in response to vascular plant diversification (Fig. 4C). These microbial decomposers may have consumed vast lignocellulose reservoirs in Carboniferous mires, contributing alongside climatic and geological factors to the sharp decline in coal accumulation after the Late Carboniferous peak (ca. 323–252 Ma) [85]. As the Earth’s only known anaerobic archaeal lignin degrader, this *Bathyarchaeia* lineage dominates modern anoxic sedimentary environments (Fig. 2B and [45]). This implies its ancestral dominance in Carboniferous mire sediments and a persistent contribution to lignocellulose degradation during deep burial processes. *Bathyarchaeia* likely outpace white-rot fungi in shaping coal preservation and long-term organic carbon sequestration, revealing the co-evolution among *Bathyarchaeia*, early plants and palaeoenvironment through metabolic innovation in lignin synthesis and degradation.

Although traditionally regarded as restricted to natural subsurface settings, *Bathyarchaeia* have been recently detected in host-associated environments, including vertebrate guts [42] (particularly ruminants) and manure-impacted systems [53, 86]. Besides, some lineages within order *Baizomonadales* also dominate gut archaeal communities in wood-feeding higher termites [41]. These findings indicate the physiological adaptation of *Bathyarchaeia* to gastrointestinal niches in plant-feeding hosts, suggesting their potential symbiotic roles in lignocellulose digestion. In addition, recent reports of *Bathyarchaeia* in landfills [87], canals [44] and microplastics [88] reflect their metabolic plasticity to emerging anthropogenic contaminants. Collectively, the niche expansion of *Bathyarchaeia* across host-associated and anthropogenic habitats highlights that ongoing metabolic innovation in decomposing evolving organic polymers may be pivotal for their 3.3-billion-year evolutionary success on Earth (Fig. 4B).

## Conclusion and outlook

This review synthesizes current understanding of *Bathyarchaeia*, emphasizing that their full phylogenetic diversity and global abundance, particularly in the deep subsurface biosphere, remain underestimated. Expanded sampling and deeper sequencing are expected to further refine their taxonomic resolution and ecological niches. Their metabolic versatility, including anaerobic degradation of complex polymers such as lignin, potential methane and alkane metabolism, and autotrophic carbon fixation, highlights their central roles in global carbon cycling by bridging terrestrial and marine ecosystems. The evolutionary history of *Bathyarchaeia* reflects deep-time interactions with geological processes and vascular plants, while their ongoing adaptation to host-associated and anthropogenic environments illustrates an ongoing co-evolution with the Earth system.

Future research on *Bathyarchaeia* should focus on several priorities: (i) developing isolation and cultivation strategies to enable further physiological and metabolic studies; (ii) applying stable isotope tracing and metabolic flux analysis [89] to elucidate their metabolic and regulatory mechanisms, particularly in lignin degradation and alkane metabolism; (iii) integrating big omics data with AI-driven modeling to quantify their contribution to global carbon sequestration and environmental effects on Earth over geological timescales; and (iv) leveraging lineage-specific lipid biomarkers (e.g. BDGTs) to develop paleoenvironmental proxies and trace their activity in subsurface ecosystems. Together, these efforts will illuminate not only the biology and evolution of *Bathyarchaeia* but also their enduring ecological impact on Earth’s carbon cycle and biosphere–geosphere co-evolution.

## Supplementary Material

Supplementary_Table_1_wraf259

## Data Availability

The datasets analyzed in Fig. 2B are available in the supplementary information and are generated from the sandpiper website (https://sandpiper.qut.edu.au/).
